# Cross-sectional associations between the screen-time of parents and young children: differences by parent and child gender and day of the week

**DOI:** 10.1186/1479-5868-11-54

**Published:** 2014-04-23

**Authors:** Russell Jago, Janice L Thompson, Simon J Sebire, Lesley Wood, Laura Pool, Jesmond Zahra, Deborah A Lawlor

**Affiliations:** 1 Centre for Exercise, Nutrition & Health Sciences, School for Policy Studies, University of Bristol, 8 Priory Road, Bristol BS8 1TZ, England; 2 School of Sport, Exercise and Rehabilitation Sciences, University of Birmingham, Birmingham B15 2TT, England; 3 MRC Integrative Epidemiology Unit at the University of Bristol, Oakfield House, Oakfield Grove, Bristol BS8 2BN, England; 4 School of Social and Community Medicine, University of Bristol, Canynge Hall, Whiteladies Road, Bristol BS8 2PS, England

## Abstract

**Background:**

Greater time spent screen-viewing (SV) has been linked to adverse health outcomes. The aim of this study was to examine whether parental SV time is associated with child SV time on week and weekend days.

**Methods:**

Cross-sectional survey of 1078 children aged 5–6 and at least 1 parent. Child and parent SV was reported for weekday and weekend days. Logistic regression examined whether parental SV time was associated with child SV time, with separate analyses for mothers and fathers and interaction terms for child gender.

**Results:**

12% of boys, 8% of girls and 30% of mothers and fathers watched ≥2 hours of TV each weekday. On a weekend day, 45% of boys, 43% of girls, 53% of mothers and 57% of fathers spent ≥2 hours watching TV. Where parents exceeded 2 hours TV-watching per weekday, children were 3.4 times more likely to spend ≥ 2 hours TV-watching if their father exceeded the threshold with odds of 3.7 for mothers. At weekends, daughters of fathers who exceeded 2 hours watching TV were over twice as likely as sons to exceed this level. Evidence that parent time spent using computers was associated with child computer use was also strongest between fathers and daughters (vs. sons) (OR 3.5 vs. 1.0, p interaction = 0.027).

**Conclusions:**

Strong associations were observed between parent and child SV and patterns were different for weekdays versus weekend days. Results show that time spent SV for both parents is strongly associated with child SV, highlighting the need for interventions targeting both parents and children.

## Background

Greater time spent screen-viewing (watching television (TV), using computers, tablets and smartphones, and playing video games) has been associated with an increased risk for cardiovascular disease, type 2 diabetes and all-cause mortality among adults [1]. Among older children and adolescents greater time spent screen-viewing (SV) has been associated with increased risk of obesity, [2] psychological difficulties [3] and metabolic risk [4]. There is little information about levels of SV and associations with disease risk factors among younger children. Since there is evidence that SV behaviours track from childhood to adulthood [5], limiting SV behaviours during early childhood may be important for long-term disease prevention.

Parents exert considerable influence on the SV patterns of young children [6] and interventions to change the latter will therefore necessitate engaging with parents. Understanding the association between time spent in SV of parents and their children is important for designing interventions [6-12]. Previous studies have reported strong associations between the TV viewing behaviours of children and their parents, with some evidence of different associations for weekdays and weekend days [13]. In a sample of 210 children aged three- five and their parents, children with a parent who watched at least two hours of TV per day were over five times more likely to do the same [7]. However, with the exception of the study outlined above, a key feature of the current literature is the focus on older children and maternal TV viewing [7,13,14]. More information is needed about the associations between parent and child SV and whether associations differ by parent and/or child gender or day of the week, and also whether associations differ by SV devices other than television. This study addressed these limitations with a specific aim of examining whether parental SV time is associated with child SV time on week and weekend days.

## Methods

**Figure 1:**
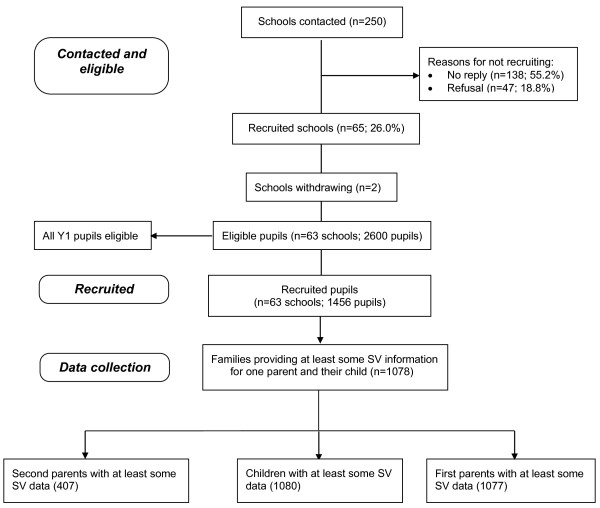
Study flow of participants.

The current analyses used data from a cross-sectional study (B-ProAct1v) conducted at the University of Bristol which aimed to identify key factors associated with physical activity (PA) and SV among children in their second year of schooling (known as Year 1 in the UK - children aged five to six). Between January 2012 and July 2013, 250 primary schools within a 45 minute drive of the University were invited to participate in the study. Over half (138 - 55%) failed to respond, and a further 47 (19%) declined. Of the 65 schools which consented to participate, two withdrew (one due to issues with low English literacy of parents and one following a change in school management) before any children had been recruited. All children in Y1 (or Reception and Y1, or Y1 and Y2 in schools with combined classes) in the remaining 63 schools were eligible to take part. Written parental consent was obtained for both the parents’ and child’s participation, and data collection took place shortly after each individual school had been recruited. The study was approved by the School for Policy Studies Ethics committee at the University of Bristol. Overall, 1456 from a potential 2600 pupils (56%) were given consent for inclusion in the study. Results here are based on data for the 1078 families that provided SV data for at least one parent and the target child. Consistent with the STROBE guidelines, Figure 1 provides a detailed presentation of the participants [15].

Parents were asked to provide information about their SV time (TV, computer, games console and smartphone) on weekdays and weekend days. The parent designated as the ‘first’ parent (i.e. the parent who completed the main questionnaire) was also asked to provide the same information, with the exception of smartphone use, on behalf of their child. Separate questions were asked for the following SV items: TV, computer/laptop, games console, tablet and smartphone (except for time spent texting or talking, and assessed for parents only). For each item the parent was asked to report the time s/he spent and their child spent using it for (a) a normal weekday and (b) a normal weekend day, with response options: none; 1 minute- 30 minutes; 31 minutes-1 hour; 1–2 hours; 2–3 hours; 3–4 hours; 4 hours or more. If the second parent was participating, they self-reported SV time separately using the same items. The assessment of TV viewing using parental response to a single question has been shown to correlate moderately (r = 0.60) with 10 days’ of TV diaries among young children [16].

TV viewing behaviour in adults and children was dichotomized based on whether the participant met the American Academy of Pediatrics (AAP) guidance of under two hours of TV per day [17]. Information for weekend days was treated similarly. Parental computer use on weekdays and weekend days was divided into two categories (less than 31 minutes per day, 31 minutes or more per day). Children’s use of computers was dichotomized into ‘no use’ and ‘some use’. Games console use amongst children was similarly categorized, as was parental use of smartphones. Insufficient numbers of adults reported using games consoles to warrant creating categories for use in subsequent analyses.

Parents’ body mass index (BMI = kg/m^2^) was calculated using self-reported height and weight. Child height was measured to the nearest 0.1 cm using a SECA Leicester stadiometer (HAB International, Northampton). Weight was recorded to the nearest 0.1 kg using a SECA 899 digital scale (HAB International, Northampton). BMI was calculated and converted to a UK age and gender specific standard deviation score (BMI z-score) using the Stata ‘zanthro’ command [18,19]. Weight categories of children were also derived using the same command. Participant postcodes were used to calculate index of multiple deprivation (IMD) scores where a higher score indicates a greater level of deprivation.

### Statistical analysis

For the purpose of analysis a male adult was assumed to be a father and a female adult a mother. To be included in the analysis, participants had to provide information about their own and their child’s SV, as well as full information for the variables used in the fully adjusted analysis model. Student t-tests were used to investigate any differences in the characteristics of participants who were included in the analyses and those excluded due to incomplete questionnaire data.

Logistic regression models were used to investigate whether parental SV time for each screen type on weekdays and weekend days (the two analysed separately) were associated with child time spent on the same screen and also associations of total SV time between parents and children. As a very small proportion of parents used games consoles (95 fathers and 41 mothers; 11% overall) their use was not explored further. Each model was adjusted for child’s gender and BMI-z score, age and BMI of parent and household IMD. In addition, the model was adjusted for whichever parent (mother or father) had reported the child’s SV time in all analyses, as it is reasonable to assume that a parent who tends to underestimate their own SV time is likely to underestimate their child’s by a similar amount. Initial analyses used all participants, followed by subgroup analysis by child gender. We subjectively compared the magnitude of associations between child gender subgroups by examining the point estimates and we also tested statistically for evidence of heterogeneity (difference in magnitude of association between the subgroups) by including the interaction term of parent SV* child gender. Confidence intervals (CI) were based on robust standard errors which took account of the clustering of participants within schools. All analyses were performed in Stata version 12.0 [20].

## Results

Data from both parents was obtained for 356 (36%) of the total of 990 children included in the analysis. Children excluded because of missing data had higher IMD scores, indicating greater levels of deprivation, than those included (Table 1). Similarly those who were excluded had higher BMI-z scores than those who were included and this was driven by a difference between the two groups of girls rather than between the two groups of boys. However, once the children’s weight was categorised, both excluded girls and boys were more likely to be overweight than children who were included in the analysis. Mothers and fathers who were excluded had higher IMD scores compared with those included. Excluded fathers were younger than those included in analyses.

**Table 1:** Characteristics of participants included in the overall analysis vs. those who are excluded

	**Included**			**Excluded**			**Difference**		
	**n**	**Mean**	**SD**	**n**	**Mean**	**SD**	**Mean**	**95% CI**	**p**
**Fathers**									
Age (years)	493	40.0	5.8	52	36.6	6.2	-3.5	-5.1 to -1.8	<0.001
BMI score	493	26.2	3.9	55	26.8	3.7	0.61	-0.49 to 1.7	0.276
IMD score^a^	493	12.4	10.2	81	16.3	13.1	3.9	1.4 to 6.4	0.002
**Mothers**									
Age (years)	793	37.4	5.5	63	36.1	5.5	-1.4	-2.8 to 0.05	0.058
BMI	793	25.1	4.5	72	24.5	4.4	-0.52	-1.6 to 0.57	0.346
IMD score^a^	793	14.4	12.2	158	21.1	16.2	6.7	4.5 to 8.9	<0.001
**Children**									
Age (years)	990	6.0	0.43	309	6.0	0.43	0.007	-0.04 to 0.06	0.786
BMI-z score^b^	978	0.23	0.93	293	0.38	1.00	0.15	0.02 to 0.28	0.016
IMD score^a^	990	14.0	12.0	181	20.2	15.9	6.2	4.2 to 8.2	<0.001
**Boys**									
Age (years)	520	6.0	0.43	144	6.0	0.23	-0.003	-0.08 to 0.07	0.941
BMI-z score^b^	512	0.25	0.96	135	0.34	0.95	0.09	-0.10 to 0.27	0.350
IMD score	520	14.3	12.2	94	19.0	15.4	4.7	1.8 to 7.5	0.001
**Girls**									
Age (years)	470	6.0	0.43	165	6.0	0.16	0.02	-0.05 to 0.08	0.627
BMI-z score^b^	466	0.21	0.90	158	0.42	1.1	0.21	0.04 to 0.38	0.014
IMD score	470	13.7	11.8	87	21.6	16.4	7.9	5.0 to 10.8	<0.001
**Weight categories**	n	Normal weight (%)^c^	Overwt (%)	Obese (%)	n	Normal weight (%)^c^	Overwt (%)	Obese (%)	
All children	978	87.1	10.0	2.9	293	78.2	17.4	4.4	0.001
Boys	512	90.4	7.2	2.3	135	83.7	14.1	2.2	0.042
Girls	466	83.5	13.1	3.4	158	73.4	20.3	6.3	0.019

^a^A high value indicates greater deprivation; ^b^Child BMI was standardized for age; ^c^Including underweight.

TV viewing was similar for mothers and fathers, with just over two-thirds reporting that they watched less than two hours per weekday (Table 2). At the weekend, however, over half (57% of fathers, 53% of mothers) watched more than two hours per day. Similarly, a higher proportion of children watched more than two hours TV on each weekend day compared with weekdays, although for all days, children were more likely to meet the guidelines than adults. Reported computer use was similar for boys and girls on weekdays and weekend days, although a higher proportion of children had higher use at weekends. Boys’ use of games consoles was greater than that of girls on all days, although use was higher for both genders at weekends than on weekdays. Parental smartphone use was similar during the week and at weekends, but was consistently higher amongst fathers than in mothers.

**Table 2:** Screen viewing levels in children and parents

	**Weekday**								
	**Male**				**Female**				
	**<2 hrs**		**2 hrs or more**		**<2 hrs**		**2 hrs or more**		**p**
	n	%	n	%	n	%	n	%	
Parent TV	342	69.4	151	30.6	556	70.1	237	29.9	0.778
Child TV	459	88.3	61	11.7	431	91.7	39	8.3	0.073
	≤30 mins		31 mins or more		≤30 mins		31 mins or more		
	n	%	n	%	n	%	n	%	
Parent PC	167	33.9	325	66.1	326	41.2	466	58.8	0.010
	Not used		Some use		Not used		Some use		
	n	%	n	%	n	%	n	%	
Child PC	200	38.5	319	61.5	182	39.0	285	61.0	0.888
Child games console	243	46.9	275	53.1	310	66.2	158	33.8	<0.001
	Not used		Some use		Not used		Some use		
	n	%	n	%	n	%	n	%	
Parent smart phone	145	29.5	346	70.5	312	39.4	480	60.6	<0.001
	**Weekend day**								
	**Male**				**Female**				
	**<2 hrs**		**2 hrs or more**		**<2 hrs**		**2 hrs or more**		**p**
	n	%	n	%	n	%	n	%	
Parent TV	212	43.1	280	56.9	376	47.5	416	52.5	0.125
Child TV	285	54.8	235	45.2	269	57.6	198	42.4	0.377
	≤30 mins		31 mins or more		≤30 mins		31 mins or more		
	n	%	n	%	n	%	n	%	
Parent PC	114	23.2	377	76.8	81	10.2	711	89.8	<0.001
	Not used		Some use		Not used		Some use		
	n	%	n	%	n	%	n	%	
Child PC	132	25.5	386	74.5	117	25.0	351	75.0	0.862
Child games console	157	30.4	360	69.3	243	51.8	226	48.2	<0.001
	Not used		Some use		Not used		Some use		
	n	%	n	%	n	%	n	%	
Parent smart phone	143	29.1	348	70.9	314	39.6	479	60.4	<0.001

There was strong statistical evidence that when fathers exceeded two hours of TV per weekday their children were 3.4 (95% CI: 1.8 to 6.7) times more likely to exceed the recommended amount of two hours per day compared with children whose fathers watched less than two hours per day. Results were similar using mothers’ TV viewing time and neither was markedly altered by adjustment for parental age and BMI, child’s gender and neighbourhood deprivation score (Table 3). There was no evidence that associations between either mothers’ or fathers’ TV viewing on weekdays with their child’s time spent TV viewing on weekdays differed between sons and daughters.

**Table 3:** **Logistic regression examining associations between parent TV and child viewing time**^**a**^

	**All children**		**Sons**		**Daughters**		
**Weekday**	N	OR [95% CI]	N	OR [95% CI]	N	OR [95% CI]	P for heterogeneity^b^
Fathers: Model 1^c^	487	3.4 [1.8 to 6.5]	267	2.8 [1.1 to 7.2]	220	4.7 [1.7 to 13.2]	0.496
Fathers: Model 2^d^	487	3.4 [1.8 to 6.7]	267	2.7 [1.0 to 7.0]	220	6.0 [2.3 to 15.7]	0.407
Mothers: Model 1	784	3.9 [2.5 to 6.1]	401	4.0 [2.1 to 7.8]	383	3.7 [1.6 to 8.6]	0.895
Mothers: Model 2	784	3.7 [2.3 to 5.7]	401	3.8 [2.0 to 7.2]	383	3.4 [1.4 to 8.3]	0.858
**Weekend**							
Fathers: Model 1	486	4.5 [3.0 to 7.0]	266	3.4 [2.0 to 5.8]	220	7.0 [4.1 to 12.2]	0.035
Fathers: Model 2	486	4.8 [3.2 to 7.3]	266	3.8 [2.2 to 6.4]	220	7.9 [4.5 to 14.0]	0.049
Mothers: Model 1	781	4.7 [3.7 to 6.1]	401	4.1 [2.7 to 6.2]	380	5.6 [3.7 to 8.5]	0.355
Mothers: Model 2	781	4.7 [3.6 to 6.1]	401	4.0 [2.7 to 6.2]	380	5.4 [3.5 to 8.4]	0.382

^a^>2 hrs. vs 2 hrs or less.

^b^Testing that associations are different in daughters and by sons; tested by adding an interaction term (parent exposure variable*child gender) into the regression model.

^c^Model 1: Unadjusted association.

^d^Model 2: Adjusted for child’s BMI-z score parent’s age, parent’s BMI, and household IMD.

The associations between parent and child TV viewing were different for weekdays and weekend days. For a weekday the children were 3.4 (95% CI: 1.8 to 6.7) times more likely to exceed the two hour threshold if their father watched TV for at least two hours per day while for a weekend day the odds were 4.8 (95% CI: 3.2 to 7.3). There were similar differences for mothers (3.7 vs. 4.7). For fathers only there was evidence that the association of time spent watching TV at weekends was more strongly associated with time spent watching TV by daughters rather than sons (OR 7.9 vs. 3.8; p_interaction_ = 0.049).

Evidence suggested that time spent using computers by fathers and mothers during the week was positively associated with child time spent using a computer during the week, but with fathers only at weekends (Table 4). During the week, there was no difference in associations between either parent for sons and daughters, whereas at weekends daughters of fathers who spent more than 30 minutes per day using a computer were 3.5 times as likely to engage in some computer use compared with daughters of fathers who spent less than half an hour per day using a computer. This was in contrast to the relationship between fathers and sons where there was no evidence of any association for computer use at weekends and an interaction term suggested that there was evidence that these associations were different for girls and boys (p_interaction_ = 0.027).

**Table 4:** Logistic regression predicting whether parental PC use predicts any PC use by child

	**All children**		**Sons**		**Daughters**		
**Weekday**	N	OR [95% CI]	N	OR [95% CI]	N	OR [95% CI]	P for heterogeneity^a^
Fathers: Model 1^b^	485	2.2 [1.5 to 3.3]	265	1.7 [1.0 to 2.8]	220	3.0 [1.6 to 5.6]	0.157
Fathers: Model 2^c^	485	2.2 [1.5 to 3.3]	265	1.7 [1.0 to 2.7]	220	3.1 [1.7 to 5.7]	0.130
Mothers: Model 1	781	1.7 [1.3 to 2.4]	400	1.7 [1.1 to 2.6]	381	1.8 [1.1 to 2.9]	0.852
Mothers: Model 2	781	1.7 [1.2 to 2.4]	400	1.7 [1.1 to 2.6]	381	1.8 [1.1 to 2.8]	0.853
**Weekend**							
Fathers: Model 1	483	1.8 [1.1 to 2.9]	264	1.1 [0.59 to 1.9]	219	3.4 [1.5 to 7.4]	0.029
Fathers: Model 2	483	1.8 [1.1 to 2.9]	264	1.0 [0.57 to 1.9]	219	3.5 [1.6 to 7.7]	0.027
Mothers: Model 1	782	0.78 [0.43 to 1.4]	400	0.57 [0.23 to 1.4]	382	1.0 [0.48 to 2.2]	0.317
Mothers: Model 2	782	0.77 [0.44 to 1.4]	400	0.56 [0.22 to 1.4]	382	0.98 [0.47 to 2.0]	0.339

^a^Testing that associations are different in daughters and by sons; tested by adding an interaction term (parent exposure variable*child gender) into the regression model.

^b^Model 1: Unadjusted association.

^c^Model 2: Adjusted for child’s BMI-z score parent’s age, parent’s BMI, and household IMD.

## Discussion

The study results show that 12% of boys and 8% of girls aged five to six watched more than two hours of TV on a weekday while 30% of fathers and mothers exceeded this threshold. On a weekend day 45% of boys and 42% of girls spent more than two hours watching TV, with 57% of fathers and 53% of mothers exceeding this threshold. A greater proportion of parents used a computer for more than 31 minutes on a weekend day than a weekday and a greater proportion of children used a games console on the weekend than a weekday, indicating differences in both parent and child SV on weekdays versus weekend days. If either mothers or fathers spent more than two hours per day watching a TV on a weekday, children were at least 3.4 times more likely to spend more than two hours watching TV. Estimates for mothers and fathers were similar. For weekend TV viewing there was an interaction between child gender and male parent TV viewing with boys 3.8 times more likely to exceed the two hour recommendation, rising to 7.9 times for girls. There was some evidence that parental weekend computer use was associated with child computer use but only in relation to girls and fathers. Collectively, these findings provide evidence of positive associations between parent and child SV time but suggest that patterns differ for weekend and weekend days. Associations are stronger for parental use at weekends than weekdays and fathers’ weekend TV and computer use may be more strongly associated with daughters’ than sons’ use.

The data reported here extends previous literature in this area which has largely focussed on mothers, young children and TV viewing. A study in Greece reported that 32% of children aged three to five spent more than two hours per weekday watching TV with both maternal (r = 0.27) and paternal (r = 0.20) hours of weekday TV correlated with child viewing time, with similar patterns for weekend days [21]. In a sample of 750 UK families with a six to eight year old child, the child was 7.8 times more likely to watch more than two hours of TV per weekday if the parent did the same; this paper did not report separately for weekend days [14]. The findings reported here are therefore comparable to previous literature but greatly extend the evidence by highlighting how patterns may differ for weekend versus weekdays. Interestingly, recent qualitative data from six European countries indicated that parents of children aged four to six reported that their children enjoyed watching TV and that most parents were not concerned about their child’s TV viewing [22]. Efforts to change behaviour are therefore likely to require improving parental understanding of why high SV is a concern and how parents’ own SV behaviour may affect their child’s. Based on the results of this study it is not possible to determine whether strategies that focus on both children and the parents are needed to change children’s SV habits. However, intuitively it seems that integrated interventions which work with both children and parents have greater potential to change behaviour than parent only focussed interventions.

The results presented here show that SV levels are higher among children at the weekend, and children with parents who engage in high levels of SV at the weekend are more likely to engage in this behaviour. Stronger associations have also been found between fathers and their daughters, compared with sons, for weekend TV and computer use. In general, time spent in SV by both parents was positively and strongly associated with their child’s SV and the magnitudes of association with both genders combined were similar. This highlights the importance of engaging both parents in the development of any interventions to reduce child SV. Many studies have struggled to engage fathers in child-focussed research [23-25] and we are not aware of any study that has specifically attempted to engage fathers in reducing child SV.

The AAP guidance on youth SV was based on expert opinion in 2001 [17] and at that time applied only to TV viewing. Although this guidance was amended in 2011 to take account of changes in technology and viewing patterns, it does not distinguish between weekday and weekend SV [26]. Although the evidence presented might suggest that greater effects could be obtained by intervening on weekend days, there are more weekdays. Thus, a child with 5 hours SV each weekend day and 2 hours each weekday spends the same amount of time in total SV during the week as at the weekend (10 hours). If the time spent each day at the weekend was double that of weekdays (e.g. 6 vs. 3 or 5 vs. 2.5 hours), for most plausible examples the total time spent SV at weekends would be less than on weekdays. Furthermore, there is no evidence that interventions to reduce SV in parents (and consequently in children) would be more effective if they solely or largely targeted weekend SV. It should also be stressed that the evidence base for greater SV in children or adults being causally related to adverse health outcomes is weak and there is currently no evidence for risk increasing at any particular threshold [27]. This is why the UK’s four Chief Medical Officers recently issued public health guidance that children and young people should limit SV but did not recommend a specific threshold [28]. Thus, consistent strategies to reduce SV in children across the whole week are likely to be needed.

### Strengths and limitations

The major strength of this study is the assessment of SV-time for both mothers and fathers, which has facilitated the examination of both maternal and paternal associations with child SV. The availability of information directly reported by fathers is a unique and important addition to the literature and addresses the current over-reliance on maternal reports of SV behaviour. Information on a key age group, for both weekdays and weekend days on a range of SV behaviours will also greatly inform the future development of targeted interventions. There are, however, limitations that should also be acknowledged. Firstly, the data is reliant on parental report of child SV. Although we are not aware of any alternative, reliable means of collecting this data, as the children are too young to self-report, a degree of error is likely in the parental reports. Of particular importance is the potential correlation of errors between the parent’s self-reported SV and their reported estimate of their child’s SV. An attempt was made to address this by adjusting for whichever parent had reported their child’s SV and it is somewhat reassuring that associations are similar for both parents. Secondly, we did not assess multi-SV in which children or parents simultaneously use multiple screen devices, which limits our ability to capture this modern pattern of viewing [10]. Finally, the cross-sectional nature of our data precludes any interpretation of the direction of association between parent and child SV. However, due to the age of the children it seems unlikely that children are influencing parent behaviour.

## Conclusions

This study shows that SV patterns of children aged five to six and their parents are different for weekdays and weekend days, with higher levels of SV on an average weekend day than an average weekday for both children and their parents. Results show that time spent SV of both fathers and mothers is strongly associated with child time spent SV, highlighting the need for interventions targeting both parents and children.

## Competing interests

The authors declare that they have no competing interests.

## Authors’ contributions

RJ, SJS, JLT and DAL were involved in the design of this study and in seeking funding for it. RJ, LP and JZ were responsible for the study conduct with LP managing data collection. LW performed all analyses. RJ wrote the first draft of the paper and coordinated contributions from other co-authors. All authors made critical comments on drafts of the paper. All authors read and approved the final manuscript.
